# Bilateral Thoracic Paravertebral Nerve Blocks for Open Gastrostomy in Patients with Amyotrophic Lateral Sclerosis

**DOI:** 10.7759/cureus.10014

**Published:** 2020-08-25

**Authors:** Arun Kalava, Tilman J Chambers, Jenna F Hoffman

**Affiliations:** 1 Anesthesiology/Pain Medicine, Breakthru Acute Pain & Ketamine Clinic, Tampa, USA; 2 Anesthesiology, University of South Florida, Morsani College of Medicine, Tampa, USA; 3 Anesthesiology, Teamhealth Anesthesiology, Tampa, USA

**Keywords:** amyotrophic lateral sclerosis, thoracic paraverteberal nerve block, epidural, gastrostomy tube, ropivicane, lidocaine

## Abstract

Most amyotrophic lateral sclerosis (ALS) patients require enteral feeding, thus necessitating placement of a gastrostomy tube (G-tube) for worsening dysphagia. This case report discusses the use of bilateral thoracic paravertebral nerve blocks (TPVB) in two patients with ALS to facilitate an open G-tube placement to avoid ventilatory assistance.

## Introduction

Approximately 70% of amyotrophic lateral sclerosis (ALS) patients develop swallowing difficulty and require a gastrostomy tube (G-tube) for enteral feeding [1]. The majority of these G-tubes are placed percutaneously under radiological guidance or open surgery [2]. When planning the anesthetic care of ALS patients, avoiding general anesthesia is desirable due to abnormal responses to muscle relaxants [3].

## Case presentation

Two patients with ALS were scheduled to undergo open surgery for G-tube placement. The first patient was a 68-year-old female with a body weight of 52 kg and a height of 160 cm in whom a G-tube could not be placed in the interventional radiology suite because her colon was located between the abdominal wall and stomach. As a result, the patient required open surgery for placement of the G-tube. The second patient was a 64-year-old female with a body weight of 70 kg, a height of 157 cm, and a peripheral oxygen saturation of 88% on room air who had undergone open gastric bypass surgery 14 years ago. Open surgical placement of the G-tube was chosen because the stomach was small and positioned cephalad in the chest cavity which would have made percutaneous placement very difficult, if not impossible. In order to avoid general anesthesia, bilateral thoracic paravertebral nerve blocks (TPVB) were performed preoperatively in the block room at thoracic levels T6-T9 for both patients. 

Preoperatively, the anatomic approach was used to perform TPVB [4]; 2% lidocaine was used for superficial anesthesia, 2 mL and 3 mL aliquots of 1% ropivacaine were bilaterally injected into the paravertebral spaces at T6, T7, T8, and T9 for a total of 20 mL. An additional epidural catheter was placed at the T7-T8 vertebral interspace after.

Intraoperatively, the first patient received 70 mg of propofol titrated in boluses of 10 mg IV over the 32 min surgery. The second patient received 2 mg of midazolam for anxiolysis and supplementary sedation, with additional epidural administration of 5 mL of epidural 2% lidocaine over the 65 min surgery. The epidural was in place for the full duration of both procedures. 

Postoperative pain scores were 0/10 in both patients. Both patients were admitted for observation. In patient 2, patient-controlled thoracic epidural analgesia with 0.2% ropivacaine at an infusion rate of 6 mL/h and self-administered 2 mL bolus with a lockout interval of 30 min was used. The first patient was discharged on postoperative day (POD) 1 and the second patient was discharged on POD 2. 

## Discussion

The total cumulative dose of ropivacaine was slightly higher than recommended [5]. In this particular case, the authors believe that the risk of providing general anesthesia was much higher than the occurrence of local anesthetic systemic toxicity.

In the second patient, an epidural was better suited to provide any additional coverage involving multiple dermatomes compared to TPVB catheters and limit opioid analgesia [6].

Open gastrostomy has been reported being performed under local anesthesia and sedation with fentanyl in patients with ALS and in older, frail subjects using subcostal transversus abdominis plane (TAP) blocks [7,8]. Both local anesthetic infiltration and subcostal TAP blocks provide somatic analgesia but not analgesia of the peritoneum, necessitating respiratory depressing analgesics like fentanyl. To avoid this, targeting the spinal nerve roots corresponding to the dermatomes involved is required [9]. Recently TPVB was used for percutaneous radiological placement of G-tube in patients with ALS and was part of the rationale behind using TPVBs in surgical dermatomes and limit respiratory depressing drugs [10]. Paravertebral blocks (PVB) for ALS was again described separately in a retrospective study with interventional radiology; G-tubes were placed by percutaneous placement under PVB in 66 cases between 2013 and 2017, and between the age range of 28-66 with a median age of 66. Mean postanesthesia care unit stay was 2.3 hours and readmission rate 4% at both one and 30 days postprocedure indicating a low rate of adverse events [11]. The results of these studies reiterate how PVB has shown to be a promising alternative to general anesthesia for these patients in a wide range of ages. 

## Conclusions

Bilateral TPVBs were reasonably adjunct in the patients reported, thus enabling open gastrostomy to be performed with minimal sedation and avoiding general endotracheal anesthesia and mechanical ventilation. We believe this is the first description of the use of bilateral TPVBs to facilitate an open G-tube placement.
